# Who adapts to whom: technology or older adults? Mechanisms of technology anxiety among older AI users

**DOI:** 10.3389/fpsyg.2026.1725814

**Published:** 2026-02-03

**Authors:** Peng Ji, Xiaoyu Liu

**Affiliations:** 1 School of Literature and Media, Shanxi College of Applied Science and Technology, Taiyuan, Shanxi, China; 2 Faculty of Humanities and Social Sciences, City University of Macau, Macao, Macao SAR, China

**Keywords:** age-friendly design, artificial intelligence, digital inclusion, older users, technology anxiety

## Abstract

**Introduction:**

As AI rapidly permeates diverse social domains, technology related anxiety among older adults during adaptation, particularly in the context of AIGC, has become a major barrier to digital inclusion. This study aims to systematically uncover the generative mechanism and hierarchical transmission pathway of older adults’ AI technology anxiety and to derive intervention implications.

**Methods:**

A mixed methods design was adopted. First, in depth interviews were conducted with 36 older AIGC users, and 14 core categories were derived using grounded theory. Second, an integrated analysis using Interpretive Structural Modeling (ISM) and Cross Impact Matrix Multiplication Applied to Classification (MICMAC) was performed to identify the hierarchical structure of influencing factors and their driving and dependence relationships.

**Results:**

ISM revealed a clear hierarchical transmission pathway. Technology anxiety is directly triggered by surface factors including insufficient AI literacy, physiological functional limitations, and technological complexity. It is transmitted through intermediate factors and ultimately driven by the deep rooted factor of social ageism. MICMAC further identified cognitive decline, social ageism, and basic resource barriers as high driving and low dependence independent factors. Insufficient AI literacy and technological complexity were categorized as high dependence surface factors whose improvement relies on systemic interventions.

**Discussion and conclusion:**

The findings demonstrate a multi level mechanism in which deep structural forces shape surface level anxiety experiences, suggesting that training or interface optimization alone may be insufficient. Coordinated interventions across policy guidance, inclusive technology design, and community support network development are proposed to help reduce the older adult digital divide.

## 1 Introduction

Against the backdrop of accelerating global population ageing, improving older adults’ quality of life has become a central societal issue. The United Nations projects that by 2050 the share of the global population aged 65 and above will rise to 16%, while the proportion of the working-age population that sustains long-term care systems is trending downward, creating severe structural pressure (United Nations, 2022). In this context, digital technologies are widely viewed as an important enabling tool for alleviating the burden on healthcare and social care and for supporting healthy ageing (Li et al., 2025). In January 2024, China’s State Council issued the Opinions on Developing the Silver Economy and Enhancing Older Adults’ Well-being, explicitly underscoring the strategic importance of leveraging technological innovation to improve older adults’ well-being (State Council of the People’s Republic of China, 2024).

Among various digital technologies, artificial intelligence (AI) is regarded as a key enabling technology for achieving these goals, given its fundamental advances in environmental perception, intent understanding, autonomous decision making, and content generation. Specifically, in health management, AI can interpret data and provide personalized health guidance (Meng et al., 2022; Thirunavukarasu et al., 2023). In cognitive support and everyday living, AI can streamline information access and assist with complex decision making (Ramalingam et al., 2020). In creative empowerment, AI can substantially lower the barriers to creation for non expert users (More, 2024). In natural interaction and emotional companionship, social chatbot technologies are also becoming increasingly mature (Brandtzaeg et al., 2022). Therefore, the systematic application of AI to ageing related domains can holistically empower multiple dimensions, including health management, cognitive support, social participation, and safety and companionship, and is emerging as a frontier direction and a critical enabler for achieving active ageing.

Although the potential utility of AI has been widely acknowledged, actual adoption among older adults remains persistently low (Yi Hoque and Sorwar, 2017; Lythreatis et al., 2022). Studies indicate that older adults often hold more conservative or negative attitudes toward new technologies and their social implications (Fernández-Ardèvol and Rosales, 2017; Nimrod, 2018; Pirhonen et al., 2020). The very features that make AI powerful may impose unprecedented cognitive, emotional, and skill related challenges on older users who are experiencing age related decline (Erhong Sun and Xuchun Ye, 2024). The resulting technology anxiety not only directly hinders technology use and weakens the improvements in quality of life and autonomy that these technologies are intended to deliver, but may also trigger a range of mental health problems (Xi et al., 2022).

Existing research on older adults’ interactions with AI has primarily focused on usage behaviors related to basic digital literacy or the use of specific functions and applications (Meng et al., 2022; Wang et al., 2024). However, within the human computer interaction paradigm, the generative mechanisms of AI related technology anxiety experienced by older users during interaction, as well as the multidimensional network of driving factors, have not yet been systematically elucidated. Therefore, clarifying why and how older users develop technology anxiety toward AI tools can help resolve the practical paradox of high technological potential but low user adoption.

Accordingly, this study adopts a mixed methods framework. First, grounded theory is used to conduct in depth interviews with 36 older users to identify the constituent dimensions of AI related technology anxiety. Next, an interpretive structural model (ISM) is employed to analyze the causal network among these dimensions. Finally, Cross-Impact Matrix Multiplication Applied to a Classification (MICMAC) analysis is applied to determine the dynamic relationships between driving factors and dependent factors, thereby systematically revealing the formation mechanism and structural relationships of AI related technology anxiety among older users. The findings are expected to provide empirical evidence and theoretical guidance for promoting inclusive AI design, advancing equitable distribution of digital benefits, and preventing the widening of the digital divide in ageing societies.

The remainder of this article is organized as follows. Section 2 reviews the literature. Section 3 describes the research design integrating grounded theory, ISM, and MICMAC analysis. Section 4 presents model development based on grounded theory. Section 5 reports the ISM hierarchical analysis and the MICMAC driving and dependence relationship analysis. Section 6 summarizes the findings and discusses contributions and limitations.

## 2 Literature review

### 2.1 Technological interaction between older adults and AI

AI technologies have become deeply embedded across multiple domains of social production and everyday life, continuously reshaping business models and daily practices. In e commerce, AI supports personalized recommendations and intelligent shopping assistance services (Soni, 2020). In healthcare, AI has been widely applied to the early diagnosis of diseases (Shen et al., 2019). Social media platforms leverage algorithms to optimize user interaction and content distribution mechanisms (Furman and Seamans, 2019). As global population ageing accelerates, the potential value of AI in promoting older adults’ health, social connectedness, and independent living has attracted increasing attention (Sun et al., 2025). Research suggests that AI can help address common later life challenges such as loneliness, cognitive decline, and deterioration in physical functioning (Shandilya and Fan, 2022; Yang et al., 2025). For example, AI enabled conversational assistants can provide emotional companionship (Ramalingam et al., 2020); assistive robots or smart home devices can help maintain cognitive and physical functioning (Mostajeran et al., 2020); and a range of intelligent tools can support the completion of activities of daily living (Ramalingam et al., 2020). These interventions aim to alleviate social isolation and enhance older adults’ autonomy and lived experience. Related research in Japan indicates that its social care system has deployed AI equipped robots or virtual agents to provide regular conversations and information exchange for older adults living alone. Such interventions may help maintain cognitive functioning and show potential as preventive measures for dementia (Igarashi et al., 2024).

Beyond material needs, older adults’ strong desire for emotional companionship, social interaction, and psychological support has become increasingly salient. This demand is intensified by changes in family structure and greater population mobility, often leading to heightened loneliness and reduced social integration (Wang et al., 2024). However, compared with younger groups, older adults generally have limited knowledge of AI technologies and fewer opportunities for direct exposure to them (Holder et al., 2018). A survey of more than 2,000 respondents in the UK shows that among those aged 55 and above, over 74% reported substantially less knowledge of and contact with AI-related information and products than younger people (Holder et al., 2018). Research based on a German sample similarly indicates that a lack of technological knowledge is a key factor influencing whether older adults adopt AI devices. Insufficient knowledge may lead more older adults to discontinue use, thereby further widening skills gaps (Kniepkamp et al., 2025).

Neurocognitive research also suggests that when dealing with open-ended tasks, older adults’ working memory capacity declines by an average of about 23% compared with younger adults (Braver and West, 2011). This may expose them to higher cognitive load when using AI tools that require complex instructions. Meanwhile, the interplay between technology and cultural cognition creates additional barriers. Older users may interpret technology-related dependence on younger people, resulting from limited digital skills, as a personal inadequacy. They may also feel psychological pressure about becoming a burden to their children (Duque and Otaegui, 2023). In addition, the algorithmic “black-box” nature inherent in AI systems renders decision-making processes opaque. This further undermines older adults’ trust in AI-generated content (Barredo Arrieta et al., 2020).

Nevertheless, existing studies have largely focused on the functional effectiveness of specific AI tools. Systematic investigation remains limited regarding how older adults, in a holistic sense, perceive, understand, and experience AI as a general-purpose enabling technology. Current age-friendly design efforts in the AI field also tend to remain at the surface level, for example, enlarging font sizes, while the underlying interaction logic is still deeply rooted in a youth-centered design paradigm (Vaportzis et al., 2017). As a result, such designs fail to genuinely address older adults’ core needs in cognition, emotion, and trust. They may even reinforce fear and avoidance of technology (Nimrod, 2018; Li and Wei, 2025). Therefore, the specific difficulties older adults encounter when using AI, their underlying emotional concerns, and how these factors dynamically shape their intentions and behaviors warrant systematic examination.

### 2.2 Technology anxiety among older users

The theoretical roots of technology anxiety can be traced to the self-efficacy mechanism in social cognitive theory (Bandura, 1997). It is commonly defined as a negative psychological state in which users hold unfavorable perceptions of their own ability to use technological tools (Meuter et al., 2005). When individuals over-focus on perceived deficiencies in their technological competence and continually anticipate failure, they may fall into a vicious cycle of depleted cognitive resources and behavioral avoidance (Deng et al., 2014). This mechanism has been widely validated in technology adoption research across contexts such as e-learning, information and communication technologies, and assistive robots (Lee and Xiong, 2022; Heerink et al., 2010; Chen and Chan, 2014). From early evidence that computer anxiety directly and negatively affects perceived ease of use (Venkatesh, 2000), to more recent findings in health information technology showing that technology anxiety leads older users to refuse mobile health devices (Meng et al., 2022), research consistently indicates that anxiety can distort individuals’ cognitive schemas about technology and systematically reduce both usage intention and exploratory behavior (Sun, 2025a; Sun, 2025b).

Older adults are more prone to technology anxiety due to natural age-related changes in physiological and cognitive functioning. Gerontological research shows that aging-related declines in cognitive resources and motor functioning (Gunasinghe and Nanayakkara, 2021) can be further amplified in AI contexts. Even in high-resource languages, LLMs still exhibit substantial biases in content generation (Ji et al., 2023), which may create cognitive confusion for older users who lack algorithmic literacy. Neuroimaging studies confirm that older adults need to recruit about 30% more prefrontal cortex activation than younger adults when processing emerging technologies (Cabeza et al., 2018). If they hold negative self-perceptions about aging and believe that physical and mental decline hinders technology learning, they are more likely to avoid new technologies (An et al., 2024). In addition, upward comparison pressure from younger generations, ageism related to technology use, and lower self-efficacy can further intensify negative emotions toward technology (Köttl et al., 2021; Mariano et al., 2022).

Different theoretical paradigms provide complementary explanations for the mechanisms underlying older adults’ technology anxiety, while also revealing their respective limitations. For clarity, Table 1 summarizes how mainstream technology acceptance and behavioral theories conceptualize this issue.

**Table 1:** Mainstream theoretical models and their explanatory emphases on older adults’ technology anxiety.

Theoretical model	Core constructs	Explanatory emphasis	References
Technology acceptance model (TAM)	Perceived usefulness Perceived ease of use Use attitude	Views anxiety as a negative emotion, reflecting worry or unease about the prospect of using technology. It hinders technology adoption by directly lowering perceived ease of use.	Davis (1989), Venkatesh (2000), Phang et al. (2006), Ryu et al. (2009), Mariano et al. (2022)
Unified theory of acceptance and use of technology (UTAUT)	Performance expectancy Effort expectancy Social influence Facilitating conditions	Conceptualizes anxiety as anxious or emotional reactions toward using the system, which affect perceived usefulness and perceived ease of use.	Venkatesh et al. (2003), Heerink et al. (2010), Powell et al. (2012)
Social cognitive theory (SCT)	Self-efficacy Outcome expectancy	Defines anxiety as fear or worry experienced when considering or actually using technology. It reflects emotional concern about unfavorable outcomes.	Bandura (1997), Peral-Peral et al. (2020), Cooper-Gaiter, (2015)

Existing research also reflects a core debate: does older adults’ technology anxiety mainly stem from insufficient individual self-efficacy, or from structural exclusion embedded in sociotechnical systems? On the one hand, studies grounded in SCT emphasize the key mediating role of self-efficacy and view anxiety as a psychological outcome of collapsing confidence when individuals face technological challenges (Chen and Chan, 2014). Evidence suggests that older adults’ motivation to adopt and adapt to advanced technologies depends primarily on three factors: first, perceived usefulness and potential of the technology; second, digital literacy that enables them to experience technological benefits; and third, the extent of personal worries about using digital technologies (Shandilya and Fan, 2022). Many older adults’ reluctance to adopt new technologies is not driven by cost, but by an insufficient understanding of the value technology may provide (Melenhorst et al., 2006; Casacuberta and Gutiérrez-Rubí, 2010). This perception is in turn grounded in digital literacy, namely an individual’s ability to identify the purposes of a technology and to use it comfortably (Shandilya and Fan, 2022). Due to cognitive aging and skill limitations, older adults are more likely to develop the belief that “I cannot master it,” which can trigger anxiety and avoidance.

On the other hand, research from critical gerontology and the sociology of technology argues that an overemphasis on individual psychology constitutes a responsibility-attribution bias, obscuring deeper forms of structural age-based exclusion. Technological complexity and technological risks are major sources of technology-related stress for older adults (Li and Xie, 2025). Pervasive ageism, skewed resource allocation, and a design paradigm that defaults to young digital natives collectively construct an older-unfriendly sociotechnical system (Ayalon et al., 2021). When using technology, older adults often experience anxiety, fear, loss of control, and limited understanding of service functions (Loveys et al., 2022; Wong et al., 2025; Sun, 2025a; Sun, 2025b). Accordingly, older adults generally report higher levels of technology anxiety than younger adults (Powell et al., 2012). Such anxiety may make them focus more on technological shortcomings, lower their evaluations of perceived usefulness, and thereby constrain both the breadth and depth of use (Wang et al., 2024).

Although prior research across multiple domains has examined the pathways through which technology anxiety exerts its effects, most conclusions about adoption motivations and barriers have been derived from non-AI technologies. As AI becomes increasingly embedded in a wide range of products, the forms that older adults’ technology anxiety takes, its specific relationships with technology adoption and use behaviors, and especially the new interaction challenges and anxiety-generation mechanisms introduced by AI, all remain to be systematically investigated in greater depth.

## 3 Research design

### 3.1 Research methods

**Figure 1:**
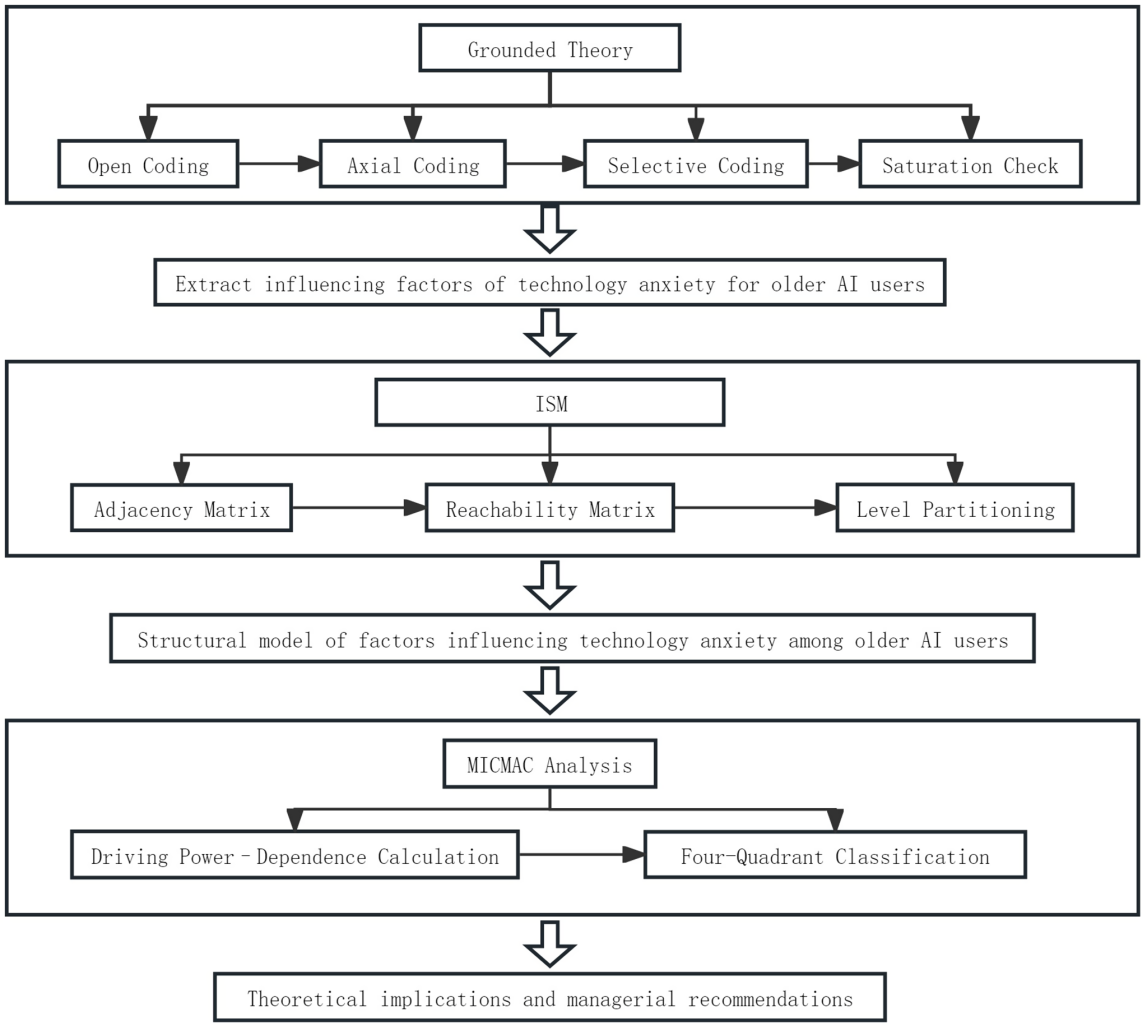
Step-by-step diagram of the study design.

This study adopts a mixed-method approach that integrates Grounded Theory with ISM-MICMAC hierarchical analysis and is conducted in three phases. First, based on interview data, Grounded Theory is applied for qualitative analysis to systematically summarize and identify the key influencing factors of technology anxiety among older AI users. Second, an ISM model is constructed to examine the causal relationships among these factors. Third, MICMAC analysis is used to quantify the driving power and dependence characteristics of each factor, identify the core driving factors, and assess the model’s rationality and validity. The specific research design procedures are shown in Figure 1.

### 3.2 Data collection

Semi structured interviews were used to collect the primary data. First, a pilot interview guide was developed by integrating insights from the existing literature with the practical application of AI technologies. Five older adults with experience using AI technologies were then recruited for pilot interviews to understand their user experiences and to obtain feedback on the guide. Based on their responses and suggestions, the interview questions and structure were refined, resulting in the final interview protocol.

The interview covered participants’ personal information, the frequency and intensity of AI technology or platform use, and their usage experiences and perceptions. To obtain richer data, the interviews used open ended questions, such as: “Through what channel did you first hear about or encounter AI tools, and can you describe the specific situation at that time?” “Have you ever felt anxious or uneasy when using or attempting to use AI technologies?” “Do you find AI tools complex to operate, and are there any aspects that confuse you?” “When problems occur while operating AI tools, what physical reactions do you experience, such as a faster heartbeat or sweating?” “Have you ever sought help from your children or friends after setbacks in using AI technologies, how do they usually help you, and is that help effective?” “Do you trust the content generated by AI technologies or the recommendations they provide?” “When you feel anxious while using AI technologies, what do you usually do to relieve these feelings?” “In what ways would you like AI tools to become easier to understand or use?”

To ensure the relevance and accuracy of the study, we applied the following criteria when selecting interview participants. First, participants were over 60 years old and had experience using or having tried AI-related products. Second, participants agreed to audio recording and permitted the storage and subsequent use of the data. Third, no restrictions were imposed on gender, region, education, or other characteristics, to ensure sample diversity and credibility. Based on these criteria, and using a combination of purposive sampling and snowball sampling, this study ultimately recruited 36 older users and completed in depth interviews with them. The respondents’ demographic information is shown in Table 2.

**Table 2:** Basic analysis of respondents (*N* = 36).

Indicator	Category	Count	Percentage (%)
Gender	Male	21	58.3
	Female	15	41.7
Age	60–69 years	21	58.3
	70–79 years	15	41.7
	80 + years	0	0
Occupation	Retired corporate employee	12	33.3
	Retired teacher	7	19.4
	Retired lawyer	3	8.3
	Retired physician	2	5.6
	Retired civil servant	4	11.1
	Retired accountant	2	5.6
	No stable occupation	6	16.7
Education	Associate degree or below	26	72.2
	Bachelor’s degree	8	22.2
	Master’s degree	2	5.6

Interviews were conducted using a combination of online and offline formats, with each interview lasting 30 to 45 min. During the interviews, the researcher adjusted the pace and prompts in response to participants’ answers to encourage authentic expression and to obtain deeper information. To reduce the impact of individual cognitive bias on the analysis, the research team carefully reviewed the interview data and conducted categorical coding. Potential interpretive deviations were addressed through rechecking interview records and consulting experts, thereby ensuring analytical accuracy. To improve the transparency of the saturation assessment, after completing open coding, axial coding, selective coding, and preliminary theory development for the first 24 interviews, the remaining 12 interviews were retained as a holdout sample for the saturation check. The criterion was that, within the existing codebook and category framework, additional data no longer generated new codes or category attributes and did not lead to substantive adjustments to relationships among categories (Guest et al., 2006).

## 4 Construction of the technology anxiety model for older AI users based on grounded theory

NVivo was used to conduct a systematic analysis of the raw corpus. Through three level coding, the data were thoroughly organized and conceptually summarized. Coding continued until theoretical saturation was reached, and an influencing factor model of technology anxiety among older AI users was ultimately developed.

### 4.1 Open coding

As the first step in category extraction in Grounded Theory, this study followed the Grounded Theory coding procedures and organized two independent coding teams to conduct open coding on 24 randomly selected interview transcripts. Each team was led by an expert professor to ensure the professionalism of the coding work. To ensure scientific rigor in the coding process, the two teams conducted cross checks and discussions after completing each one third of the coding tasks. If the coding results were largely consistent, they were accepted directly. If disagreements existed, further discussion continued until coding consistency reached 90 percent.

During coding, the researchers tried to preserve the original form of the corpus as much as possible and directly quoted participants’ original expressions. For example, the statement “I also want to learn, but every time my child tries to teach me, they have no patience to explain it clearly” was classified as “insufficient intergenerational support.” The statement “Isn’t this AI tool the same as Baidu or Sogou?” was classified as “comprehension barriers.” The statement “What is a model? What are prompt words? We cannot learn these new things” was classified as “declining learning ability.” Open coding produced a large number of initial concepts, with overlaps among categories. After data cleaning steps such as merging similar items and removing invalid items, 35 valid initial concepts were identified. Through relational analysis and cluster organization of the initial concepts, 15 independent categories were ultimately refined, including declining cognitive ability, insufficient AI literacy, and behavioral inertia lock in. Detailed results are shown in Table 3.

**Table 3:** Results of open coding.

Excerpts from raw data	Initial concepts	Free nodes
I just learned how to use Doubao (an AI tool) a few days ago, but today I totally forgot the steps.	Memory deterioration	Cognitive decline
What’s a ‘model’? What are ‘prompts’? We cannot grasp these newfangled terms.	Impaired learning capacity	
Isn’t this AI stuff the same as Baidu or Sogou?	Comprehension barriers	Deficiency in AI literacy
My son installed tons of AI apps for me, but they are just collecting dust now.	Operational difficulties	
I cannot tell whether this news was written by AI or by real journalists.	Information discrimination impairment	
No matter how perfect AI-generated photos appear, they lack the soulful warmth of the fading prints in my ancestral camphorwood chest.	Dependence on established habits	Behavioral inertia lock in
How could AI poems compare to Li Bai’s? Humans will always surpass machines!	Low novelty acceptance	
Those tiny buttons keep popping up; my eyes ache from squinting.	Visual acuity decline	Physiological functional limitations
The robotic voices of AI assistants are too muffled to understand.	Hearing impairment	
I accidentally click on ads all the time; it’s so easy to mis-tap!	Tactile hyposensitivity	
To generate an AI image, I have to pick models, adjust sliders, write prompts, then tweak filters—five layers of menus! Impossible to remember.	Operational complexity	Technological complexity
It feels suspicious; a single button click gives instant results, like some black-box trickery.	Algorithmic black box	
The app updated again right after I learned it. I cannot keep up with this rat race.	Rapid technological iteration	
Why are there fifty buttons on one screen? I do not know where to start!	Interface complexity	Unfriendly interaction design
Zero instructions. I’ve fiddled for hours and still cannot make it work.	Lack of guidance	
My accented Mandarin confuses the voice AI; half my commands get lost in translation.	Voice interaction barriers	
Every AI response follows the same robotic template: ‘Firstly… Secondly… In conclusion…’	Information homogenization	Content quality risks
Most AI videos online are deepfakes; you cannot trust what you see anymore.	Information ethics	
The AI dumps walls of text on me; no highlights, no summaries.	Information overload	
Everyone assumes old folks and tech do not mix; it’s become a stereotype.	Negative stereotypes	Societal age bias
People keep saying “you cannot teach an old dog new tricks,” and it gets in your head.	Competence denial	
Our village WiFi can barely load basic Ernie Bot; forget about AI art tools!	Technological diffusion inequality	Foundational resource barriers
The AI class at the senior college started with “neural networks” jargon; total gibberish to us.	Resource misallocation	
If my child taught me patiently, I would be willing to learn as well.	Intergenerational support	Emotional support
If my old friends are learning, I will learn along with them.	Peer support	
If the community could offer AI classes specifically for older adults, I would definitely go every day.	Community support	Resource support
I’d never take medical advice from an algorithm; who knows if it’s safe?	Content distrust	Technology distrust
This feels scammy, like there’s something shady under the hood.	Algorithmic distrust	
What if it steals my home address or bank passwords? The paranoia is real.	Privacy security distrust	
Just hearing ‘artificial intelligence’ gives me a migraine.	Psychological rejection	Technophobia
My hands shake and my heart races every time I open these apps; pure stress.	Physiological tension	
The world belongs to AI and the youth now. We’re being digitally retired.	Self-negation	Technology related inferiority
My grandson edits photos with AI in minutes, but I’m too slow to learn.	Intergenerational comparison disparity	
I’ll use AI only if someone holds my hand through it; otherwise, why bother?	Restricted usage	Technology avoidance
Those AI charts give me panic attacks; I’ve deleted all the apps for good.	Refusal to use	
Why learn fancy AI commands when Baidu gets me answers faster?	Technological downgrading	

### 4.2 Axial coding

Axial coding, as the subsequent step after open coding, aims to further analyze the relationships among independent concepts and ultimately cluster them into main categories. Through coding and clustering, this study generated 15 independent categories. After relational and comparative analysis, six core main categories were formed: user factors, technological factors, environmental factors, social support, manifestations of technology anxiety, and consequences of technology anxiety. The complete mapping relationships of the coding results are shown in Table 4.

**Table 4:** Results of axial coding.

Core categories	Subcategories	Category definitions
User factors	Cognitive decline	Age related deterioration in information processing capacity, affecting the understanding and operation of AI technologies
	Deficiency in AI literacy	Lack of the knowledge base and skills required to use AI tools, resulting in a digital divide
	Behavioral inertia lock in	Path dependence formed through past experience, hindering exploration of and adaptation to new technologies
	Physiological functional limitations	Natural decline in sensory systems and motor functions, creating physiological barriers to technology use
Technological factors	Technological complexity	Design features of AI tools that exceed older users’ cognitive load
	Unfriendly interaction design	Non inclusive design that fails to consider characteristics of older users, increasing difficulty of use
	Content quality risks	Issues in the reliability, ethicality, and information density of AI generated content
Environmental factors	Societal age bias	Stereotypes and negative labels toward older adults’ use of new technologies
	Foundational resource barriers	Difficulties in effectively accessing or using AI tools due to problems in resource distribution and provision
Social support	Emotional support	Psychological and emotional support older adults receive while learning to use AI technologies
	Resource support	Tangible external resources older adults receive while learning to use AI technologies
Manifestations of technology anxiety	Technology distrust	Distrust of AI tools regarding generated outputs, algorithm transparency, and related aspects
	Technophobia	Physiological and psychological stress responses when encountering or using AI tools
	Technology related inferiority	Self worth negation and social identity crisis triggered by technological setbacks
Consequences of technology anxiety	Technology avoidance	Choosing to limit use, refuse use, or switch to traditional platforms to obtain information

### 4.3 Selective coding

**Figure 2:**
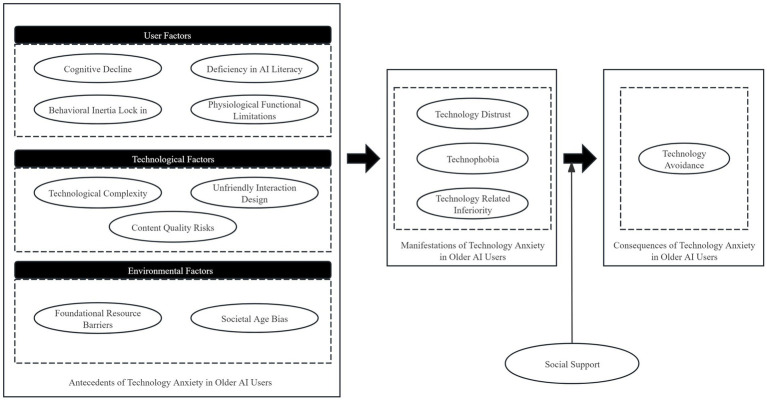
Research model and analytical framework of technology anxiety factors among older AI users.

Selective coding, also known as core coding, is the final stage of the three level coding system in grounded theory and is primarily responsible for theoretical abstraction and integration. After an in depth analysis of the main categories, and in light of the causal characteristics underlying older adults’ technology anxiety in AI use, this study constructed a multi level relational network model. Through triangulation using the constant comparative method and storyline analysis, the study ultimately confirmed the causal mechanisms linking the three main categories, namely user factors, technological factors, and environmental factors, to manifestations of users’ technology anxiety, as well as the causal mechanisms linking manifestations of technology anxiety to the consequences of technology anxiety (see Figure 2). A summary of the specific coding information is presented in Table 5.

**Table 5:** Results of selective coding analysis.

Pathway	Relationship structure	Definition of the relationship structure
User Factors → Manifestations of Technology Anxiety	Causal relationship	Personal factors such as cognitive decline, insufficient AI literacy, behavioral inertia lock in, and physiological functional limitations are internal factors influencing older AI users’ manifestations of technology anxiety
Technological Factors → Manifestations of Technology Anxiety	Causal relationship	Technological complexity, unfriendly interaction design, and content quality risks are external factors influencing older AI users’ manifestations of technology anxiety
Environmental factors → Manifestations of Technology Anxiety	Causal relationship	Societal age bias and foundational resource barriers are external factors influencing older AI users’ manifestations of technology anxiety
Manifestations of Technology Anxiety → Consequences of Technology Anxiety	Causal relationship	Technology distrust, technophobia, and technology related inferiority are important factors influencing older AI users to limit use, refuse use, or switch to traditional platforms
Moderating effect of social support on the anxiety to avoidance link	Moderating relationship	Social support can buffer the positive effect of manifestations of technology anxiety on technology avoidance. That is, higher levels of social support can weaken the behavioral withdrawal caused by anxiety

### 4.4 Saturation testing

In grounded theory research, a theoretical saturation test is a key step to ensure research quality and credibility. To verify the stability and completeness of the theory, this study conducted a follow up validation analysis using 12 reserved original texts that were not included in the initial coding. The results show that the existing theoretical framework adequately covers the characteristics of the newly emerging data, and no new explanatory categories emerged. To strengthen the validity of the verification, we invited three experts in the field of human computer interaction for in depth interviews and conducted coding analysis of the interview data. The results again confirmed that no new concepts or categories were generated. Based on the multiple forms of validation above, the grounded theory coding results of this study are confirmed to have passed the theoretical saturation test.

## 5 ISM model of factors influencing technology anxiety among older AI users

This study introduces ISM, a classic method in systems science, to explore the multidimensional causes of technology anxiety among older AI users (Warfield, 1974). Compared with traditional linear analytical approaches, ISM is well suited to analyzing the dependency and driving relationships among elements in complex systems and can construct a clear multilevel hierarchical structure. In doing so, it reveals the essential pathways and hierarchical logic underlying the focal issue (Attr i et al., 2013). This provides a strong analytical framework for systematically deconstructing and visualizing the internal network of relationships among the factors influencing technology anxiety in older AI users. The specific procedure is as follows:

### 5.1 Constructing the adjacency matrix

The adjacency matrix is used to describe the direct relationships among factors within a system. To ensure the scientific rigor and accuracy of the ISM, we designed a multistage validation procedure. First, we presented the 9 core concepts derived from grounded theory to the 36 original participants and held in depth discussions on the interrelationships among these elements, carefully incorporating their feedback and suggestions. Next, we formed an expert panel comprising four professors in human computer interaction and four doctoral researchers in information behavior. Using a five level influence scale: 0 (no influence), 0.25 (weak influence), 0.5 (moderate influence), 0.75 (strong influence), 1 (decisive influence), the panel evaluated the strength of influence among the elements while also revising potential cognitive bias with reference to user feedback. To ensure the objectivity of the matrix, a 60 percent consensus threshold was set, meaning that a relationship was considered valid only when more than 60 percent of the experts agreed on it. Ultimately, a 9 by 9 adjacency matrix was generated (Table 6).

**Table 6:** Adjacency matrix.

Element	Cognitive decline	Insufficient AI literacy	Behavioral inertia lock in	Physiological functional limitations	Technological complexity	Unfriendly interaction design	Content quality risks	Societal age bias	Foundational resource barriers
Cognitive decline	0	1	1	0	1	0	0	0	0
Insufficient AI literacy	0	0	0	0	0	0	0	0	0
Behavioral inertia Lock in	0	1	0	0	1	0	0	0	0
Physiological functional limitations	0	0	0	0	0	0	0	0	0
Technological complexity	0	0	0	0	0	0	0	0	0
Unfriendly interaction design	0	1	0	0	0	0	0	0	0
Content quality risks	0	1	0	0	0	0	0	0	0
Societal age bias	0	1	1	0	0	0	0	0	1
Foundational resource barriers	0	1	0	0	1	1	0	0	0

The matrix strictly follows Boolean operation rules. When and only when $a_{\mathrm{ij}}=1$ , element $a_{i}$ has a direct influence on element $a_{j}$ . When $a_{\mathrm{ij}}=0$ , no direct influence relationship exists. For example, the value at the intersection of the row for cognitive decline and the column for insufficient AI literacy is 1, indicating that cognitive decline directly influences insufficient AI literacy.

### 5.2 Constructing the reachability matrix

The adjacency matrix represents the direct relationships among system elements, whereas the reachability matrix reflects both direct and indirect associations among elements. In this study, the reachability matrix *M* was obtained through Boolean power operations, expressed as:


$${\left(A+I\right)}^{K-1}\neq {\left(A+I\right)}^{K}={\left(A+I\right)}^{K+1}=M$$

where *A* is the adjacency matrix, *I* is the identity matrix, and *k* is the minimum number of iterations that satisfies the convergence condition. The computation was implemented in MATLAB 2022b, and the results are shown in Table 7. The Boolean properties of the reachability matrix are as follows.

**Table 7:** Reachability matrix *M.*

Element	Cognitive decline	Insufficient AI literacy	Behavioral inertia lock in	Physiological functional limitations	Technological complexity	Unfriendly interaction design	Content quality risks	Societal age bias	Foundational resource barriers
Cognitive decline	1	1	1	0	1	0	0	0	0
Insufficient AI literacy	0	1	0	0	0	0	0	0	0
Behavioral inertia lock in	0	1	1	0	1	0	0	0	0
Physiological functional limitations	0	0	0	1	0	0	0	0	0
Technological complexity	0	0	0	0	1	0	0	0	0
Unfriendly interaction design	0	1	0	0	0	1	0	0	0
Content quality risks	0	1	0	0	0	0	1	0	0
Societal age bias	0	1	1	0	1	1	0	1	1
Foundational resource barriers	0	1	0	0	1	1	0	0	1

Row vector interpretation: $m_{\mathrm{ij}}=1$ indicates that factor *i* can influence factor *j* through a direct or indirect path.

Column vector interpretation: $m_{\mathrm{ij}}=1$ indicates that factor *j* is subject to the multistep influence of factor *i*.

Meaning of zero entries: $m_{\mathrm{ij}}=0$ indicates that no reachable path exists between the two factors.

For example, in the row for cognitive ability, the entries are 1 under the columns for cognitive ability, AI literacy, inertia lock in, and technological complexity, indicating that cognitive decline can directly or indirectly influence insufficient AI literacy, behavioral inertia lock in, and technological complexity.

### 5.3 Decomposing the hierarchical relationships among elements

When performing hierarchical decomposition, it is necessary to identify the reachability set, antecedent set, and their intersection for each element. These sets can be extracted from the reachability matrix *M*. For a given element, the reachability set *R* refers to the set of elements marked as 1 in the row corresponding to that element in *M*. The antecedent set *Q* refers to the set of elements marked as 1 in the column corresponding to that element in M. The intersection set SS is determined by comparing *R* and *Q* and identifying their common elements, that is, *S = R∩Q*. The detailed results are shown in Table 8.

**Table 8:** Reachability set and antecedent set derived from the reachability matrix *M.*

Element	Reachability set *R*	Antecedent set *Q*	Intersection set *S = R∩Q*
Cognitive decline	1, 2, 3, 5	1	1
Insufficient AI literacy	2	1, 2, 3, 6, 7, 8, 9	2
Behavioral inertia lock in	2, 3, 5	1, 3, 8	3
Physiological functional limitations	4	4	4
Technological complexity	5	1, 3, 5, 8, 9	5
Unfriendly interaction design	2,6	6,8,9	6
Content quality risks	2,7	7	7
Societal age bias	2, 3, 5, 6, 8, 9	8	8
Foundational resource barriers	2, 5, 6, 9	8, 9	9

The numbers indicate specific elements. For example, 2 denotes the second element. The number of iterations was 3.

### 5.4 Constructing the interpretive structural model

After deriving each element’s reachability set, antecedent set, and intersection from the reachability matrix, hierarchical extraction of category elements can be performed. When (*R = S*), the element can be assigned to a level. After removing the extracted elements, the above procedure is applied recursively. Iteration continues until all matrix elements are fully decomposed, yielding a multilevel hierarchical structure model.

The results show that the nine categorical elements were ultimately divided into four levels:

Level 1 (Surface layer) L1 = {Insufficient AI literacy, Physiological functional limitations, Technological complexity}

Level 2 (Intermediate layer) L2 = {Behavioral inertia lock in, Unfriendly interaction design, Content quality risks}

Level 3 (Intermediate layer) L3 = {Cognitive decline, Foundational resource barriers}

Level 4 (Root layer) L4 = {Societal age bias}.

**Figure 3:**
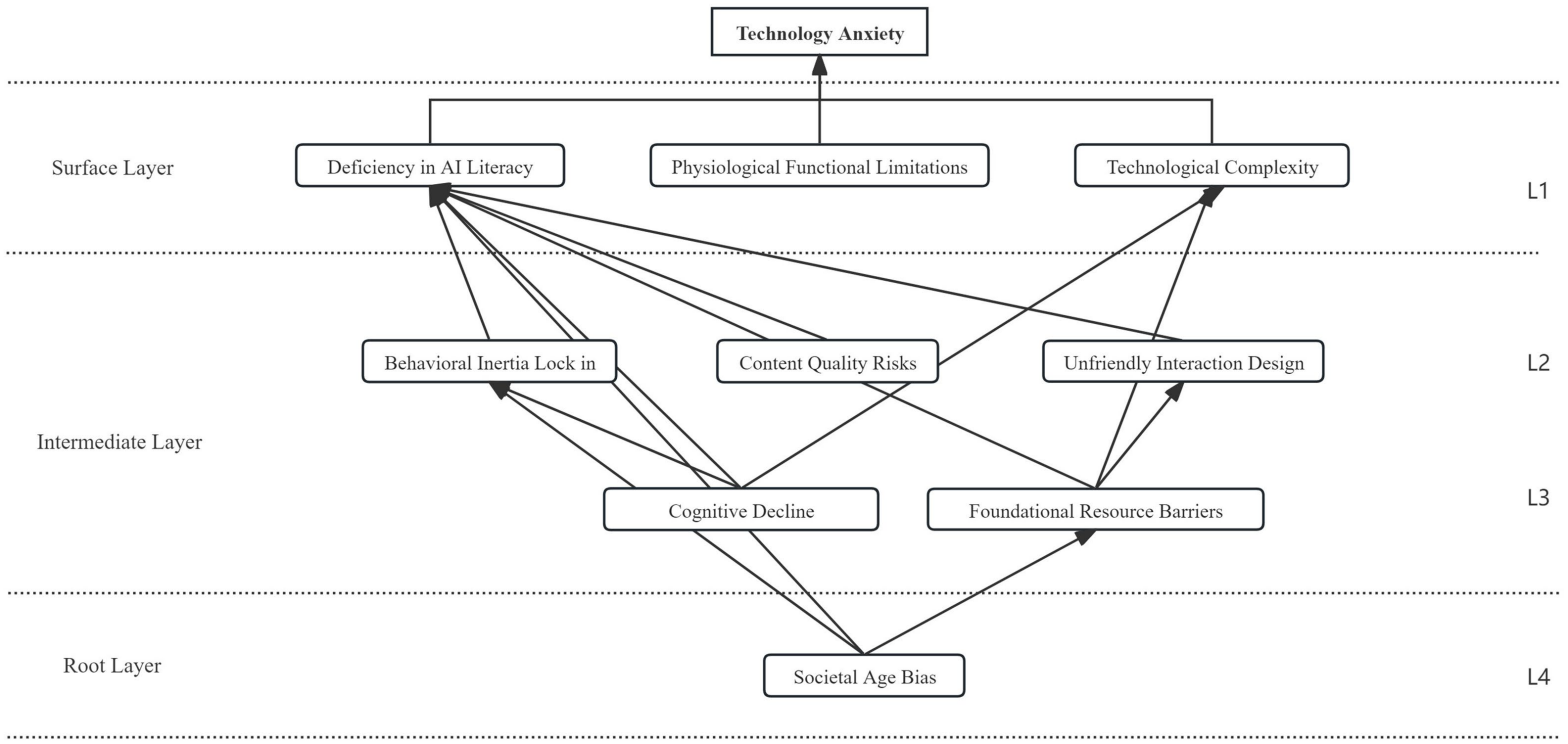
Structure and relationship analysis diagram of factors influencing technology anxiety among older AI users.

Based on the above analysis, this study constructs an interpretive structural model of factors influencing technology anxiety among older AI users, shown in Figure 3. The specific relationships and influence analysis are as follows:

The first level (L1) directly triggers the emergence of technology anxiety among older AI users, including insufficient AI literacy, physiological functional limitations, and technological complexity. These three factors constitute the immediate shock older adults experience when facing AI. Physiological functional limitations involve age-related declines in vision, hearing, and touch, which can produce a form of embodied misalignment with technology and create a sense of mismatch between the body and technological interaction. One interviewee vividly described this: “Those buttons are as small as sesame seeds. With one swipe the whole screen starts jumping around. My eyes and hands cannot keep up, and I panic right away” (Interviewee P15). At the same time, insufficient AI literacy prevents them from understanding basic concepts such as “prompts” and “models,” while technological complexity exceeds their cognitive processing speed, generating a sense of helplessness: “I simply do not know what AI is doing. I learn it today and forget it tomorrow” (Interviewee P08). According to emotion regulation theory (Gross, 2015), when individuals appraise a technological interaction as threatening and perceive their resources as insufficient, they tend to prioritize situation avoidance. In this study, this mechanism manifests in three ways. First, technology anxiety directly leads to behavioral avoidance. To end uncomfortable embodied experiences, older adults may proactively give up, for example: “As soon as I see a complicated interface my heart races, so I just uninstall it. Out of sight, out of mind” (Interviewee P22). Second, repeated setbacks lead to learned helplessness. When multiple attempts end in failure, they form a fixed belief such as “No matter how I learn, it is useless. I just cannot keep up with this era” (Interviewee P18), and stop making efforts to learn. Third, anxiety strengthens perceived burden, meaning that they restrain themselves because they worry that asking for help will become a burden to their children. Multiple interviewees noted: “I do not dare to keep asking my kids. I am afraid they will find me annoying. The more I ask, the more I feel like a burden” (Interviewees P07, P29). Together, these three points form the key psychological bridge from short term anxiety to sustained resistance.

L2 and L3 are intermediate layers and function as indirect influencing factors. They consist of cognitive decline, behavioral inertia lock in, unfriendly interaction design, content quality risks, and foundational resource barriers. As structural mediators in the formation of technology anxiety among older AI users, the intermediate layers indirectly shape the decision making process. Cognitive decline refers to reduced efficiency in information processing among older users, manifested in memory decline and weakened learning capacity. Behavioral inertia lock in reflects older users’ reliance on established habits. When the learning cost of new technologies exceeds a psychological threshold, users choose to maintain older, less efficient but familiar routines, which in turn contributes to insufficient AI literacy. Unfriendly interaction design points to a lack of age friendly design in interfaces, including excessive visual density, insufficient operational guidance, and inadequate compatibility with dialects. Consequently, after older adults encounter negative experiences during use, they continue to feel a misalignment between the body and technology, which then leads to technology avoidance. Meanwhile, uneven AI content and information quality undermines older users’ trust in AI platforms. As one participant stated, “A lot of AI generated videos are fake” (Interviewee P11). Foundational resource barriers reflect structural imbalances in the allocation of technological resources, such as urban rural gaps in digital infrastructure and shortages in the provision of age friendly learning resources. In essence, this represents age exclusion in technology governance, resulting in an intergenerational rupture in technological accessibility, reinforcing ageist cognitive schemas, and jointly constituting the social environmental pressures underlying older adults’ technology anxiety.

The fourth level (L4) is the root layer that drives the entire system, namely societal age bias. In media narratives and everyday interactions, stereotypes such as “older people are not suitable for learning new technologies” are common. In interviews, users mentioned that “People around me all say older people do not need to learn new technologies, and even if we try, we cannot learn them” (Interviewee P19), which is directly internalized as self doubt. Moreover, many AI technologies are developed primarily for younger users, and older adults’ needs are neglected from the outset of resource allocation. As one interviewee pointedly observed: “From the moment this AI came out, it was never intended for us older people to use this high tech” (Interviewee P33). This deep seated bias not only intensifies individuals’ experiences of frustration, but also systematically reproduces the barriers transmitted through the intermediate layers and expressed at the surface layer.

### 5.5 MICMAC analysis

To further investigate the interrelationships among the levels, this study employs the MICMAC analysis method to systematically quantify each factor’s driving power and dependence, revealing the core roles of different elements and their interconnection mechanisms.

The formulas for the driving power *Di* and the dependence *Rj* are as follows:


$$D_{i}=\sum_{j=1}^{n}am_{\mathrm{ij}},(i=1,2,3,\cdots n)$$

$$R_{j}=\sum_{i=1}^{n}am_{\mathrm{ij}},(j=1,2,3,\cdots n)$$

**Figure 4:**
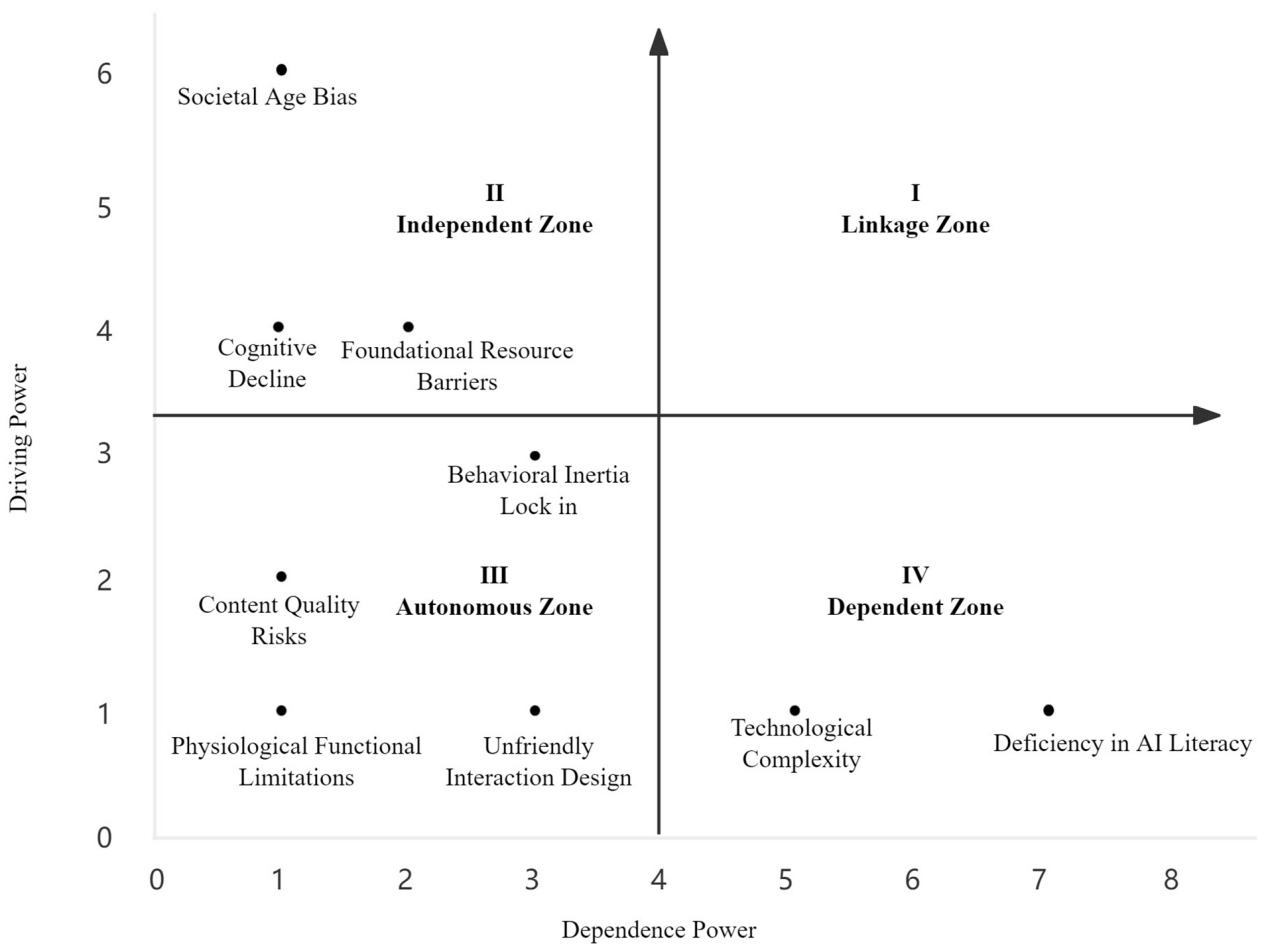
MICMAC analysis quadrants of influencing factors for technology anxiety among older AI users.

Here, $am_{\mathrm{ij}}$ denotes the element in the *i* th row and *j* th column of the reachability matrix *M*. The sum of the row vector is *Di*, which represents the total influence of the *i* th factor on other factors. The sum of the column vector is *Rj*, which reflects the total dependence of the *j* th factor on other factors. *n* denotes the dimension of the reachability matrix *M*. Based on the driving power and dependence values of each factor, a two-dimensional coordinate plot was drawn; the results are shown in Figure 4.

MICMAC analysis provides important validation and supplementation for the vertical hierarchical structure revealed by ISM from a horizontal network perspective of driving power and dependence relationships. The results indicate that the influencing factors can be classified into three categories according to their roles in the system, and these categories correspond to the ISM level distribution.

First, factors in the independent cluster, including social ageism, basic resource barriers, and cognitive decline, are characterized by high driving power and low dependence. This is consistent with the ISM results that place social ageism in Level 4 (Root layer, L4) and basic resource barriers and cognitive decline in Level 3 (Intermediate layer, L3). As key drivers of the system, they exert broad influence on other factors while being difficult to change through short term interventions. This finding confirms that technology anxiety among older adults is not a surface issue, but is rooted in deep structural exclusion and the underlying conditions of individual ageing.

Second, factors in the dependent cluster, insufficient AI literacy and technological complexity, exhibit high dependence and low driving power. This offers a profound and complementary interpretation of their positioning in ISM as factors in Level 1 (Surface layer, L1) that directly trigger anxiety. Although they are the most directly perceived barriers, their emergence and persistence at the system level are highly dependent on shaping by the independent cluster and intermediate layer factors. For example, insufficient AI literacy is constrained by cognitive capacity and educational resources, while technological complexity originates from youth centered design paradigms and operational procedures. Therefore, focusing solely on surface problems is unlikely to alleviate anxiety, and upstream driving factors must be addressed in a coordinated manner.

Finally, factors in the autonomous cluster, behavioral inertia lock in, unfriendly interaction design, content quality risks, and physiological functional limitations, show both low driving power and low dependence. This further specifies the nature of the ISM factors in Level 2 and Level 3 (Intermediate layer, L2 and L3). As hubs that transmit anxiety, they are influenced by factors in the Root layer while also directly shaping user experience, yet the systemic effort required to change them is relatively lower. For instance, unfriendly interaction design is a clearly defined technical issue that can be improved directly through inclusive design principles. This points to a feasible intervention entry point, namely using inclusive technology design to alleviate technology related embodied maladaptation caused by the interaction between physiological limitations and design deficiencies.

In sum, MICMAC analysis not only confirms the validity of the ISM hierarchy, but more importantly, from the perspective of power relationships among factors, it explains why certain deep factors in the independent cluster serve as key leverage points, whereas certain surface problems in the dependent cluster require system wide coordination to resolve. Together, the two analyses depict a complex system that is both clearly layered and dynamically interconnected, providing a reference for developing multi level intervention strategies that range from fundamental governance to localized optimization.

## 6 Conclusion

### 6.1 Theoretical contributions

First, drawing on grounded theory, this study employed the ISM-MICMAC approach to construct an influencing factor model of technology anxiety among older AI users. Unlike prior studies that rely on single pathway explanations, this research not only depicts the hierarchical transmission relationships among influencing elements, but also systematically analyzes the strategic priority of key factors, thereby forming a theoretical framework with dynamic analytical capability. The model identifies a surface triggering mechanism consisting of insufficient AI literacy, technological complexity, and physiological functional limitations, as well as an intermediate transmission mechanism represented by cognitive decline. Because these mechanisms involve universal processes of biological ageing and fundamental logics of technology design, they may have strong cross cultural explanatory power. By contrast, social ageism and basic resource barriers in the model are deeply embedded in specific socio cultural and institutional arrangements. For example, in China, social ageism often intertwines with intergenerational responsibility and filial piety norms, manifesting as internalized pressure not to burden one’s children. In individualistic cultural contexts, ageism may instead appear more directly as devaluation of individual competence or subtle exclusion from social participation. Similarly, basic resource barriers in China are often reflected in a pronounced urban rural digital divide, whereas in welfare states they may be more evident as disparities in access to digital skills training resources. Therefore, this model offers an analytical framework that can be adapted to different contexts.

Second, this study introduced biological constraints such as sensory decline and reduced motor coordination into human computer interaction analysis, successfully revealing the embodied roots of older adults’ difficulties in technology adaptation. In contrast to the tendency of traditional research to focus only on psychological perception pathways, this framework emphasizes that technology design must attend to the bodily materiality of older users. When interfaces present dense small icons or require fine long press operations, they can conflict with older adults’ bodily schemata. In such situations, anxiety does not primarily originate from a psychological fear, but from the frustration generated as the body becomes entangled with technology in awkward, effortful interaction. For instance, participants’ accounts such as “If I am not careful, I end up clicking an advertisement link” (Interviewee P10) and “Those tiny words make my eyes ache and I start to panic” (Interviewee P05) capture the immediate bodily tension and loss of control that arise when declines in visual acuity and finger motor precision collide with interface design logic. This experience resonates with the view of philosopher of technology Don Ihde that technology can become an other, forming an antagonistic and unsettling relationship with the body (Ihde, 1990). Older users’ anxiety and resistance toward AI can be understood as their bodies articulating an incompatibility with the technological world. This perspective shifts the focus of age friendly design from surface usability to fundamental respect for and accommodation of older users’ bodily materiality, offering a new ontological foundation for human computer interaction research.

Third, a key theoretical advance of this study lies in linking structural critiques of technological exclusion in later life with a subject centered empowerment perspective through the moderating effect of social support, thereby elevating the discussion of technology anxiety to the theoretical level of technological justice and digital agency. The study finds that social support from family, peers, and communities can effectively buffer the transformation of technology anxiety into behavioral avoidance. This is not merely a matter of providing help. Its deeper implication is that social support constitutes a crucial context in which older adults build digital agency. In the interviews, when older adults are situated within supportive networks, their discourse shifts from passive endurance to active exploration. For example, one participant noted, “If my old friends are all learning this AI photo album, I will learn along with them. We can ask each other, and I do not feel embarrassed” (Interviewee P24). This vividly shows that peer support provides not only information but also a safe space that reduces technology related shame and empowers individuals to try. Another participant stated, “If my child could be more patient, not think I am too slow, and teach me step by step, I would be willing to try and see what AI can actually do” (Interviewee P07). This indicates that patient and respectful intergenerational support is a starting point for activating willingness to explore and intrinsic motivation, and it serves as the soil in which agency can emerge. At the same time, this study also reveals that social ageism and basic resource barriers, as antecedent conditions in the model, point precisely to systemic technological injustice (Fraser, 2009), namely institutionalized exclusion of older adults embedded in technology design, resource allocation, and social discourse. Ageism functions as a form of symbolic violence that becomes internalized as self doubt, as one participant said, “People around me say older people do not need to learn these new things, and even if we try we will not learn them. Hearing that so often, I started to believe it myself” (Interviewee P19). Such internalized ageism erodes, at its root, older adults’ identity recognition as legitimate technological subjects (Xi et al., 2022). Resource barriers, meanwhile, represent distributive injustice that materially deprives them of equal rights to access digital opportunities. As one participant observed, “The internet in the village is poor, so even if I want to try those advanced AI functions, they will not load. In the city, the courses at the senior university start with algorithms and models. It is too advanced, and it does not match what we actually want to learn, which is simply how to use it” (Interviewee P31). Therefore, the integrated framework of this model avoids the limitation of attributing problems solely to individual deficits, while also overcoming a deterministic tendency to portray older adults as entirely passive victims. It offers a strong perspective for understanding the dialectical relationship between structural constraints and agentic practice in digital inclusion.

### 6.2 Practical implications

This study aims to resolve older adults’ technology anxiety and proposes multi level intervention strategies to advance age friendly technology.

At the level of policy guidance and support, emphasize inclusive institutional design and resource reallocation. To address the basic resource barriers and social ageism identified in this study, public policy can play a key steering role. Promote the development of encouraging AI age friendly design guidelines, such as large fonts, high contrast modes, and simplified navigation flows, and systematically integrate older adults’ digital literacy enhancement into urban and rural community service systems. In resource poor areas, support the development of simple, locally relevant brochures and offline support stations, for example “AI Healthcare Guide for Older Adults” and “AI Travel Planning for Older Adults.” Media campaigns and public discourse should also promote positive narratives of ageing and digital engagement, reducing ageist stereotypes such as digital abandonment and creating a more welcoming technological environment for older people.

At the level of technology design and development, the core is to build an experience friendly, progressively adaptive interaction environment. Age friendly technology should not be a mere reduction of features but a deep reshaping of experience. AI tool developers are advised to prioritize and optimize alternative interaction modes at the input and feedback layers, such as voice interaction, gesture control, and image recognition, to address mismatches between sensory decline and touch interfaces. At the learning pathway level, AI product designers can embed “novice guides” or “exploration modes” that allow older users to start from the simplest question and answer interactions and gradually unlock more complex functions, while providing repeatable prompts and help. AI generated content should also indicate its source, for example “This information was generated by AI,” and use plain language explanations to ease older adults’ unease about algorithmic black boxes.

Relief of technology anxiety also requires secure social contexts. Society can promote “digital buddy” programs by training volunteers or younger seniors as technology tutors who provide regular, patient guidance at senior universities, community centers, and public libraries, focusing on practical AI usage issues. Families can be encouraged to adopt a co learning support model in which children and parents explore an AI function together, such as creating a digital photo album or planning a trip, transmitting skills through concrete tasks and helping older adults build enthusiasm and confidence for learning.

### 6.3 Limitations and future direction

Despite deepening understanding of the mechanisms behind older adults’ technology anxiety through a mixed-methods approach, this study has several limitations. First, the sample is limited. All participants were primarily urban and mainly retirees. This sampling strategy allowed for in-depth exploration of a specific group but means the sample did not cover rural areas, ethnic minorities, or older adults with much weaker digital literacy. Second, the sample homogeneity limited our ability to systematically compare AI anxiety across different socio-economic subgroups. For example, retired professionals versus blue-collar older adults, and higher-educated versus lower-educated older adults, may differ significantly in technological cognition, access to learning resources, and self-efficacy, leading to divergent triggers, intensity, and coping strategies for anxiety. Future research should include more diverse samples and use comparative designs to explore the multidimensional intersections of the digital divide. Third, the methodological choices in the current study were driven by its exploratory aims. Subsequent studies can and should employ tools such as structural equation modeling to test hypotheses based on our findings, thereby advancing cumulative knowledge and methodological integration in this field. Finally, the study’s cultural specificity was not fully examined. The anxiety-generation model developed here is based on in-depth investigation of Chinese older AI users. Future research should test the weights and interactions of the model’s hierarchical factors in different cultural contexts and explore the model’s boundaries and extensions from a cross-cultural perspective.

## Data Availability

The original contributions presented in the study are included in the article/supplementary material, further inquiries can be directed to the corresponding author.
